# Occurrence of Histamine in Commercial Cat Foods under Different Storage Conditions

**DOI:** 10.3390/vetsci9060270

**Published:** 2022-06-04

**Authors:** Alberto Altafini, Paola Roncada, Gaetan Minkoumba Sonfack, Alessandro Guerrini, Gianluca Antonio Romeo, Giorgio Fedrizzi, Elisabetta Caprai

**Affiliations:** 1 Department of Veterinary Medical Sciences, University of Bologna, Via Tolara di Sopra 50, Ozzano dell’Emilia, 40064 Bologna, Italy; alberto.altafini@unibo.it (A.A.); alessandro.guerrini5@unibo.it (A.G.); 2 Reparto Chimico Degli Alimenti, Istituto Zooprofilattico Sperimentale della Lombardia e dell’Emilia Romagna “Bruno Ubertini”, Via P. Fiorini 5, 40127 Bologna, Italy; g.minkoumbasonfack@izsler.it (G.M.S.); giorgio.fedrizzi@izsler.it (G.F.); elisabetta.caprai@izsler.it (E.C.); 3 Italian Ministry of Health—Directorate-General for Animal Health and Veterinary Medicinal Products, Viale Giorgio Ribotta 5, 00144 Roma, Italy; ga.romeo-esterno@sanita.it; 4 Istituto Zooprofilattico Sperimentale dell’Abruzzo e del Molise “G. Caporale”, Via Campo Boario, 64100 Teramo, Italy

**Keywords:** histamine, pet food, cat, LC-MS/MS

## Abstract

In fish-based foods, one of the effects of inappropriate storage can be the formation of biogenic amines. Among these, histamine is considered one of the most toxic. The purpose of the present study is to assess the occurrence of histamine in fish-based pet foods, and to evaluate the changes in histamine content during storage at different temperatures. For the analysis, an LC-MS/MS method was used. Fifty-eight pet foods were purchased, and an aliquot of them was analyzed just after opening the packages. Histamine was detected in 12 samples and concentrations ranged from 1.5 to 30.1 mg/kg. The remaining of each positive sample was divided into seven subsamples. One of them was used as a control sample and kept at −20 °C, while the other six were exposed to different environmental conditions. Samples exposed to room temperature showed no significant changes in histamine levels, while samples exposed to high temperatures showed significant increases in histamine content. Finally, samples exposed to refrigerator temperature showed a slight decrease in histamine levels. Under the experimental conditions, the EU limit of 100 mg/kg established for fishery products was never exceeded. These results seem to indicate a low risk of histamine intoxication in cats fed fish-based pet food.

## 1. Introduction

The attention to the welfare of pet animals is constantly increasing. Owners tend to pamper their pets, whether it is by food (packaged industrial food for 96.97%), or the purchase of accessories and hygiene products [1]. The way of feeding pets has changed over the years; there has been a shift from a diet based on leftovers and meat scraps to the purchase of complete and balanced feed prepared by food industries in the sector. Buyers’ orientation is to choose premium, high-quality products and smaller packages [2]. The Italian pet-food market in 2020 grew both in value (+4.2% compared to 2019) and volume (+2%), with an annual turnover of more than EUR 2 billion. In the current COVID-19 pandemic crisis, pets have taken on an increasingly important role in households, which now include more than 60 million dogs, cats, small mammals, birds, fish and turtles [3]. According to the research commissioned by ASSALCO, in the last year and a half, 32.8% of those who owned one or more pets bought or adopted at least one more. Cat food represents 53.2% of the total value of the market at grocery, traditional pet shops and pet shop chains for a value of just over EUR 1.2 billion (+5.9% compared to 2019), while dog food accounts for the remaining 46.8% of the market, for a value of about EUR 1 billion (+2.3% compared to 2019) [4].

Pet foods are commercially available in three basic forms: dry, semi-moist and canned (moist). The main difference in this categorization scheme is the water content. Dry foods usually contain up to 12% water, semi-moist foods 22–35% water and canned food between 60 and 87% water [5]. They are mainly composed of meat and by-products from various animal species (usually beef, chicken and fish) and cereals (generally rice and corn), but also potatoes [6]. The main segment of the cat-food market is wet food, which covers about two thirds of the market (62.2% in value terms), and in 2020 it increased its turnover by 9.1% as compared to the previous year. Dry food makes up 32.8% of cat-food sales and grew 4.2% in value. In addition, cat snacks are a small but growing segment: in 2020, its share of the total cat-food market reached 4.9% [4].

Similar to foodstuffs for human consumption, the growing health-consciousness of the public led to an increased attention to the safety and quality of pet foods [7]. The general feed safety requirements, which also cover pet foods, can be found in Reg. (EC) No 767/2009. This regulation lays down rules on the placing on the market and use of feed for both food- and non-food-producing animals within the European Community [8].

Pet food is carefully controlled to ensure animal health. Every three years in Italy, the Ministry of Health amends and implements the National Animal Feeding Plan (PNAA) in order to guarantee, according to the provisions of Regulation (EC) n. 178/2002 and Regulation (EU) 2017/625, an official feed-control system along the entire food chain [9].

The proper storage of pet food is very important to maintain organoleptic properties (taste, odor, appearance and texture) and nutritional characteristics. Pet diet components are susceptible to microbial, chemical, physical and insect spoilage. In several cases, water activity (a_w_) value is the primary parameter responsible for food stability [10]. In fact, it is a critical factor for microbial growth, texture, flavor, chemical reactivity (such as browning or lipid oxidation) or enzyme activity. In general, the higher the water activity value, the greater the perishability of a food product. Canned foods are typically higher than 0.85 a_w_ and allow for the easy growth of bacteria, yeast and molds, while dry pet foods are in the a_w_ range of 0.40–0.45 and, at this low level, microbial stability is not an issue [7]. The effect of temperature on the water activity of food is product specific. Some products increase water activity with an increasing temperature, others decrease a_w_ with an increasing temperature, while most high-moisture foods have negligible changes in relation to temperature [11]. More than 250 different foodborne hazards have been identified, including infectious bacteria, viruses and parasites, and noninfectious chemicals and toxins [12,13]. Generally, foodborne diseases are caused by the consumption of food or water contaminated with pathogens or their toxins [14]. In fish-based foods, one of the effects of inappropriate storage and/or temperature abuse can be the formation of biogenic amines (BAs) due to microbial enzymatic activities [15].

Adverse food reactions may caused on an immunologic basis (food allergy (FA)), a non-immunologic basis (food intolerance (FI)) or may also be toxic reactions (intoxications). FA is an aberrant, adverse immune response elicited by exposure to a particular food substance, most often a protein. FI may be caused by a metabolic problem or pharmacologic reaction (it is the case of certain amino acids in spoiled fish that can be transformed into BAs by several microorganisms) or can be idiosyncratic. In dogs and cats, it may be difficult to distinguish between FI and FA because they can be associated with similar foods and gastrointestinal signs [16].

BAs are basic nitrogenous compounds of low molecular weight having aliphatic, aromatic or heterocyclic structures that are formed by the microbial decarboxylation of amino acids or by the amination and transamination of aldehydes and ketones during decomposition [17,18,19,20]. BAs can also be found in other food products, including meat, dairy, fruits, vegetables, nuts, chocolates and fermented products [17]. These compounds are resistant to heat treatment applied in food processing, and their levels can be used as indicators of the degree of freshness or spoilage of food, since BAs are absent or only present at very low levels in fresh products [21,22]. The most important BAs found in food are histamine, tyramine, putrescine, cadaverine, phenylethylamine, agmatine, tryptamine, serotonin, spermidine and spermine [23,24]. Of these compounds, the first two are considered as the most toxic and particularly relevant for food safety. The main prerequisites for the presence of BAs in foods are the availability of free amino acids, the presence of microorganisms producing BA enzymes and conditions allowing their growth and enzyme production (particularly temperature, pH and water activity) [25,26]. Histamine is present in most foods, but in greater abundance in fish and fishery products. This biogenic amine is the main component in food poisoning known as scombroid poisoning. In fact, these intoxications are related to the consumption of fish of the *Scombridae* and *Scomberesocidae* families, such as tuna, mackerel and bonito. These species contain high levels of the free amino acid, histidine, in their muscles, which is decarboxylated to histamine [24,27]. However, other non-scombroid fish, including sardine, bluefish, salmon, greater amberjack fish, swordfish, herring and trout, can cause the disease and, for this reason, it is also called histamine fish poisoning [28,29]. *Escherichia coli* is the bacterial agent most commonly implicated in these intoxications, while the other bacteria species with histidine decarboxylase activity that can be involved include *Vibrio*, *Proteus*, *Serratia*, *Enterobacter*, *Klebsiella*, *Clostridium*, *Salmonella* and *Shigella* [29,30,31]. Although some of them are present in the normal microbial flora of live fish, most seem to be derived from post-catching contamination on board fishing vessels, at the processing plant or in the distribution system, or in restaurants or homes [32]. In humans, the signs and symptoms associated with scombroid poisoning typically occur within 10 to 30 min of fish ingestion and most commonly include flushing of the face, neck, upper torso and upper back; headache; pruritus; urticaria; dry mouth, but also a burning sensation of the mouth and oropharynx; and lightheadedness. Gastrointestinal symptoms can include abdominal cramps, nausea, vomiting and diarrhea. Other reported symptoms are bronchospasm, hypotension and dyspnea [27,28,29,33,34]. Symptoms typically disappear in a few hours, but some can last up to 36–48 h [29,34].

Histamine is the only BA for which the European Union established maximum legal limits for certain fish and seafood species [35,36]. The reference standard is the Commission Regulation (EC) No 2073/2005, as amended by Commission Regulation (EC) No 1441/2007 and Commission Regulation (EU) No 1019/2013, laying down microbiological criteria applicable to foodstuffs for certain microorganisms. This regulation established that, for fishery products from fish species associated with a high amount of histidine, the mean value of histamine of 9 units comprising the sample must be ≤100 mg/kg, with no more than 2 sample units having values between 100 and 200 mg/kg. Moreover, for fishery products that have undergone enzyme-maturation treatment in brine, the above limits are doubled [37,38,39]. Considering the strong influence of Bas on food quality, the U.S. Food and Drug Administration (FDA) has set for histamine a more restrictive limit (50 mg/kg) [40]. On the other hand, with respect to pet food, to date, no guidelines have been developed on the threshold levels for biogenic amines [41]. The scientific literature reports very few studies evaluating the effect of biogenic amines on dogs and cats due to ethical reasons. However, it was found that elevated levels of biogenic amines can cause food poisoning and detrimental effects on palatability and nutrition [24]. The toxicological responses to histamine depend on the method of administration, and the toxicological effects differ among species [42]. Furthermore, in many cases, healthy adult animals have developed detoxification systems for biogenic amines, whereas puppies, as well as ill animals and breeding females, are more susceptible to the adverse effects of these substances [43]. Some authors have also reported that it is likely that many foods are also responsible for non-immunologic intolerance in cats and, in particular, certain fish that contain high levels of histamine, e.g., tuna and any dried or inadequately preserved fish [44]. Based on all the considerations made above, it is clear that the determination of biogenic amine levels is essential to ensure the quality of pet-food products. The purpose of this study is firstly to assess the occurrence and levels of histamine in canned commercial fish-based pet foods purchased in Italy from supermarkets and specialized shops. The present research also aims to evaluate the changes in histamine content during storage of pet foods at different temperature conditions for normal household use. For the laboratory determinations, a liquid chromatography-tandem mass spectrometry system (LC-MS/MS) was used.

## 2. Materials and Methods

### 2.1. Sampling

A total of 58 commercial pet-food samples were collected from supermarkets, traditional pet shops and pet-shop chains, for the most part located in the metropolitan city of Bologna (Emilia Romagna, north Italy) and its surrounding area. Each sample was numbered and registered in an appropriate product data sheet. Furthermore, pet-food packages were photographed, and their ingredient labels recorded in order to have all the available information about each product. The survey focused on cat food (which constitute more than half of the pet-food market). It was chosen to sample wet foods, which cover two-thirds of the cat-food market and are considered to be more perishable than dry foods. The presence of fish among the ingredients was another criterion of choice, because histamine can be found in greater abundance in fish and fishery products. Finally, products of 24 different brands in different price ranges were purchased. The main types were labeled as tuna products (*n* = 46). Some of them contained tuna only (*n* = 22), while others also contained different fish or fish products (*n* = 10) or even meat and meat products (*n* = 14). The remaining pet food samples, not containing tuna (*n* = 12), were based on other fish, and half of these samples (*n* = 6) also contained meat and meat products. Of the total products sampled, most were marketed as complementary foods (*n* = 35), while the others were complete foods (*n* = 23). Most products (*n* = 32) were in the high-price range (≥10 EUR/kg), while the others were in the medium- (5.00–9.99 EUR/kg) and low (≤4.99 EUR/kg)-price ranges (*n* = 14 and 12, respectively). Pet-food samples were stored at room temperature until opening. After taking a portion of the product to be analyzed for the presence of histamine, the samples were stored at −20 °C for further investigations.

### 2.2. Evaluation of Histamine Content after Storage at Different Temperature Conditions

Following the analysis, the positive samples were tested to evaluate the influence of temperature on histamine content during storage. For this aim, these samples were thawed at 4 °C and divided into 7 subsamples. One of them was used as a control sample and kept at −20 °C until analysis, while the other 6 were each exposed to a different environmental condition: room temperature for 24 h, room temperature for 48 h, high temperature (25–45 °C) for 24 h, high temperature (25–45 °C) for 48 h, refrigerator temperature (4 °C) for 5 days and refrigerator temperature (4 °C) for 10 days, respectively. The temperatures were monitored using a maximum–minimum thermometer. At the end of the test times, each sample was placed in a freezer food bag and immediately frozen at −20 °C until analysis.

### 2.3. Solvents and Reagents

The reference standard of histamine dihydrochloride was supplied by Sigma-Aldrich Co. (St. Louis, MO, USA). Methanol (LC-MS grade) was from VWR Chemicals (Milano, Italy), while formic acid (LC-MS grade) and ammonium formate (analytical grade) were from Carlo Erba Reagents (Cornaredo, MI, Italy). Hydrochloric acid 1N (analytical grade) and potassium dihydrogen phosphate anhydrous (analytical grade) were from Sigma-Aldrich Co. (St. Louis, MO, USA). Ultrapure water used throughout the experiments was produced by an Evoqua Water Technologies system (Pittsburgh, PA, USA).

### 2.4. Chromatographic Apparatus and Conditions

Histamine analyses were performed by LC-MS/MS on a chromatographic apparatus Alliance HT 2695 (Waters, Milford, MA, USA) coupled to a Quattro Ultima Platinum triple-quadrupole mass spectrometer with a electrospray ionization source (Micromass, Manchester, UK). Chromatographic separation was achieved in isocratic elution mode and at room temperature using an analytical column Luna HILIC 200 Å 150 × 2 mm 3 µm (Phenomenex, Torrance, CA, USA). The mobile phase consisted of 80% of methanol and 20% of 15 mM ammonium formate water solution pH 3.3. The flow rate was set at 0.25 mL/min and the injection volume was 10 µL. Based on the structural properties of histamine, the positive ionization modes (ESI+) were applied. The parameters were as follows: cone voltage, 40 V; capillary voltage, 3.5 kV; source temperature, 120 °C and desolvation temperature, 350 °C. Mass Lynx TM 4.0 SP4 software (Micromass, Manchester, UK) was used to control the instruments and process the data. The data acquisition was in multiple reactions monitoring (MRM) mode. The ion transitions and mass parameters monitored for histamine are reported in Table 1.

### 2.5. Sample Preparation

A 2.5 ± 0.1 g aliquot of minced sample was weighed into a 50 mL Falcon tube and 20 mL of a mixture of methanol-50 mM phosphate buffer (50:50, *v*/*v*) was added. The sample was then mixed on a horizontal shaker for 30 min and centrifuged at 10,000 rpm in a refrigerated centrifuge at 5 °C for 10 min. Five milliliters of the upper layer were transferred to an appropriate centrifuge tube. If the solution was cloudy, it was centrifuged at 3000 rpm for 5 min and the upper oily phase was removed. Finally, the solution was diluted at 1:100 (*v*/*v*) with hydrochloric acid 0.1 N and, following vortexing, an aliquot was transferred to a HPLC vial for analysis.

### 2.6. Quantification

For the quantification of histamine in pet food, calibration curves were created by first preparing a set of histamine standard solutions in solvent at different concentrations. The curves were constructed based on the peak area resulting from LC-MS/MS analysis of each standard solution. The histamine content of the unknown samples was calculated by extrapolating the peak area from the calibration curve and then multiplying the value obtained by the dilution factor (1:800) applied in the sample preparation procedure.

### 2.7. Performance Evaluation

An in-house validation was conducted to check the performance of the adopted analytical method. For this aim, the following parameters were evaluated: specificity, linearity and range, limit of detection (LOD), limit of quantification (LOQ), recovery and precision (assessed in terms of repeatability and within laboratory reproducibility). Specificity was proved using 20 blank samples, which were analyzed and evaluated for interference. Linearity was determined in the working range on the basis of the determination coefficient (R2). The working range was the range of the concentrations of histamine in the standard solutions for which the calibration curve had been plotted. LOQ and LOD were established on the basis of a signal-to-noise ratio at histamine retention times of 10:1 and 3:1, respectively. Recovery was estimated at 3 concentrations (20, 100, 200 μg/g) by comparing the peak area of histamine in spiked samples and the peak area of histamine in pure standard solutions at the same concentration levels. Repeatability was assessed by preparing and analyzing six blank samples spiked at three concentration levels (20, 100, 200 μg/g) for a total of 18 determinations, and it was expressed as the relative standard deviation (RSD%) of results. Analyses were conducted with the same instruments, on the same day, and by the same operators. Similarly, the within laboratory reproducibility was evaluated for six blank samples fortified at three concentration levels, but, in this case, the procedure was performed on three different days and by different operators, for a total of 54 determinations (18 per day), and the RSD% of the replicate measurements was calculated. Finally, quality control (QC) samples were processed and analyzed together with the unknown samples as further confirmation of the reliability of the analytical results.

### 2.8. Statistical Analysis

All the statistical computations were performed using MS Excel software (Microsoft, Redmond, WA, USA). The statistical difference between the average histamine concentrations detected in the various groups of samples was determined by using a *t*-test, and *p* value <0.05 was accepted as significant.

## 3. Results and Discussion

### 3.1. Assay Validation

**Figure 1:**
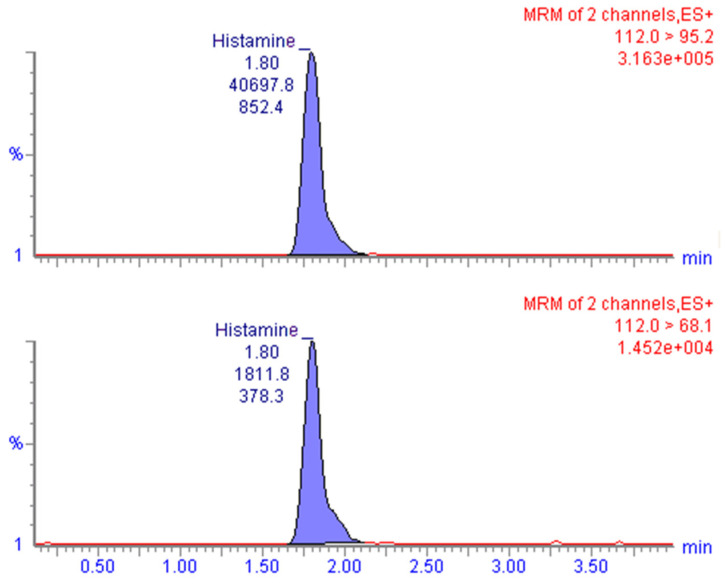
Chromatogram obtained after LC-MS/MS analysis of a naturally contaminated sample at 28.7 mg/kg level representing the quantitation (*m*/*z* 112.0 > 95.2, upper) and confirmation (*m*/*z* 112.0 > 68.1, lower) MRM transitions.

Specificity requirements were met with the applied analytical conditions. The twenty blank samples analyzed for assay interference did not show interfering peaks at the retention-time window of histamine, and no abnormal background noises were observed. The retention time of the analyte in the contaminated samples and standard solutions was about 1.8 min, while the total run time was 5.0 min (Figure 1).

All the reference curves in solvent generated from the analysis of pure histamine standard solutions showed a satisfactory linearity in the range of concentrations tested, which changed from 1 to 250 ng/mL. This range allowed us to evaluate the histamine concentrations in matrix from LOQ to 200 µg/g in consideration of the dilution factor of the extraction procedure (1:800). In all regressions, the coefficient of determination (R^2^) was always >0.99. The LOQ and LOD of the method were 1 and 0.5 µg/g, respectively, well below the histamine limit (100 mg/kg) established at the EU level for fishery products from fish species associated with a high amount of histidine. Recovery experiments were performed at 3 spike levels within the working range, and the average recovery percentages were found to be between 78.75% and 82.92%, while the overall average recovery was 80.81%. The data for the percent recoveries and the mean recoveries for each fortification level are shown in Table 2.

Intraday and inter-day tests to evaluate the repeatability and reproducibility of the analytical method performed well. The RSDs of quantification results ranged from 8.79 to 9.55% and from 8.84 to 10.43%, respectively (Table 3). Therefore, the overall results of the method validation demonstrated the reliability of the method for the determination of histamine in pet foods.

### 3.2. Occurrence of Histamine in Pet-Food Samples

In the first phase of the study, 58 samples of pet food were analyzed and 46 of them were negative, while 12 samples (20.7% of total samples) showed a concentration level of histamine ranging from 1.5 to 30.1 mg/kg. The data are reported in Table 4.

The average concentration (±standard deviation) of the positive samples was 13.2 ± 11.2 mg/kg. By grouping the samples based on the ingredients of animal origin, different data can be extrapolated (Table 5). All samples collected contained fish and/or tuna, some also contained meat. The highest percentage of positives was found among samples containing meat and tuna (35.7%, 5 out of 14). This percentage was almost the same as that among samples containing meat (35.0%, 7 out of 20), while it was lower among samples containing tuna (21.7%, 10 out of 46), only tuna (13.0%, 3 out 23) and only fish (i.e., no meat) (13.2%, 5 out 38). Samples not containing meat or tuna were all negative. Moreover, the average concentration (±standard deviation) of histamine in the positive samples containing meat was 15.4 ± 11.2 mg/kg, and was higher than that found in samples containing only fish, tuna and only tuna (10.2 ± 11.8, 11.8 ± 11.0 and 15.1 ± 13.7 mg/kg, respectively), but, due to high intra-group variability, differences among the average concentrations detected were not statistically significant (*p* > 0.05). These results seem to indicate the ingredient “meat” as a primary risk factor for this type of intoxication, while the ingredient “tuna” seems to play a minor role. The data are reported in Table 5.

When observing the samples by price range, the highest percentage of positives was found among mid-range products (35.7%, 5 out of 14), while these percentages were 15.6% (5 out of 32) and 16.7% (2 out of 12) among high-end and low-end products, respectively. Moreover, the average concentration (±standard deviation) of histamine in the mid-range positive samples was 20.5 ± 9.3 mg/kg, and was significantly higher (*p* < 0.05) than that found in the high-range positive samples (5.1 ± 4.7 mg/kg).

The average concentration of the two positive low-end samples resulted from two very different concentration values (28.7 and 2.3 mg/kg). The data are reported in Table 6.

In any case, it should be noted that none of the samples collected were found to be contaminated on a serious level, if we take as a reference the EU limit established for fishery products from fish species associated with a high amount of histidine.

After all purchased pet foods were tested for histamine, the twelve positive samples were divided into 7 subsamples, which were each exposed to a different environmental conditions, as described in Section 2.2, with the aim of evaluating the influence of temperature on histamine content during storage. The subsamples were then analyzed, and the results are reported in Table 7.

The exposure of the samples to room temperature simulated the condition in which the food was fed to the animal, but it was not consumed within 24/48 h. This situation could occur, for example, when pet owners are absent and entrust the management of the animal to an external person, or in catteries where animals can be fed once a day. Exposure of the samples to high temperatures simulated the condition in which the food can be found in the summer period when it is administered outdoors, as may occur, for example, in some feline colonies. Exposure of the samples to refrigerator temperature simulated the condition in which a food, after opening, is stored in the refrigerator for several days because the owner has forgotten about it, or in the case of large packages containing food for several meals.

Statistical analysis of the data for histamine concentrations detected in the groups of samples subjected to different storage conditions is shown in Table 8.

As shown in Table 7, between the first and second control analyses, there was a reduction in histamine content in the positive samples, which had been stored at −20 °C after the first analysis. This data agree with the findings of Ben-Gigirey et al. who reported a decrease in histamine in tuna samples frozen for several months [45]. In line with this trend, foods that showed low concentration levels in the first analysis were negative in the second analysis. Evaluations of histamine concentrations in the various groups of samples subjected to different environmental conditions were conducted, primarily with respect to the concentrations detected in the group of samples stored at −20 °C after the first analysis (control group, T_0_-B). Changes in the histamine concentrations between the different groups were also evaluated. The statistical difference between the averages concentrations was determined by using the t-test (Table 9).

Samples stored at room temperature (T_1_-A, T_1_-B) did not show significant increases of histamine. In most cases, concentrations decreased slightly after 24 h, while after 48 h the values were similar to those detected in the control samples. Otherwise, both groups of samples exposed to high temperatures (T_2_-A, T_2_-B) showed a significant increase in histamine content (*p* < 0.05). Compared to the control group, after 5 days, the increase was generalized; while, after 10 days, a further increase was detected in most cases (8 out of 12 samples). Finally, both groups of samples stored at refrigerator temperature (T_3_-A, T_3_-B) showed on average a slight decrease in histamine content, although not statistically significant (*p* > 0.05). A general slight decrease in histamine after 5 days (except in sample 53) and a further slight decrease after 10 days (with the exception of sample 13) were observed.

The variations in histamine content found in the different samples could be related to the type and degree of bacterial contamination. The decrease that occurred in some cases could be related to the prevalence of histamine-decomposing bacteria versus histamine-producing bacteria. As can be observed, even under the experimental conditions, no particularly high histamine concentrations were detected and the EU limit of 100 mg/kg established for fishery products was never exceeded. However, it must be said that the samples exposed to the experimental environmental conditions, following freezing, had a decrease in histamine content compared to the initial amount. Consequently, it can be assumed that the concentration values obtained as a result of the tests could have been higher.

The highest concentrations of histamine were found in samples subjected to high temperatures, an environmental condition in which food should never be left, but in some situations can occur, for example, in feral cat colonies. These colonies are very widespread in Italy and are officially recognized by the framework law n. 281 of 14 August 1991 as well as by regional laws on the protection of cats [46]. Hygiene and health rules for the proper management of a feral cat colony provide the administration of food at fixed times so that cats consume all the food immediately and the creation of feeding points sheltered from the weather (sun and rain). However, these requirements are not always met, especially if colonies consist of many cats or if animals are wary of humans. Feed can therefore remain in the bowl for hours, which, although sheltered from direct sunlight, in summer can be exposed to high temperatures. Moreover, eventual intoxications in these types of animals are difficult to identify. It is for this reason that, in the present study, it was considered interesting to evaluate the possible formation of histamine also in this particular environmental condition.

Recent experiments evaluating the effect of histamine on dogs and cats were not conducted due to ethical reasons. Blonz and Olcott investigated the effects of spoiled tuna containing a high level of histamine (2500 µg/g) fed to pigs, cats, dogs and rabbits. Pigs were the only animals that showed a reaction to the fish (regurgitation). The study indicated a different reactivity depending on the pig breed (78% of Duroc pigs and 33% of Hampshire pigs regurgitated within 40 min after the consumption of spoiled tuna, while no reaction was observed in Large White pigs). Furthermore, pigs weighing 18–36 kg were more reactive than the heavier animals. As for the other animals, the authors reported that 33% of dogs and 25% of cats refused to eat, a sign of the detrimental effect of histamine on the organoleptic characteristics of food [47]. A study conducted on newborn and adult dogs reported the cardiovascular effects of some biogenic amines and their precursors. The results show that tyramine, 1 to 1000 mg/kg, increases arterial pressure and heart rate to about the same extent in the puppy and the adult, while histamine, 0.01 to 100 mg/kg, has greater depressor effects in adult dogs, while no significant changes in heart rate are observed in either age group [48]. Adverse reactions to ingested scombroid fish in cats were reported by Guilford et al., who cited 6 cases of cats showing certain symptoms, such as salivation, vomiting and diarrhea, following the intake of uncooked anchovies. According to the authors, dogs may also occasionally be affected by histamine toxicosis, and they reported the case of a dog with angioedema and diarrhea after ingestion of spoiled fish [49]. Hickman et al. reported that kittens fed purified experimental diets containing histamine at 50 µg/g showed no deleterious effects [50].

Since it is quite common among canids and wildcats to occasionally feed on decomposed carcasses, they must have therefore developed adaptive mechanisms to detoxify biogenic amines. Domestic dogs and cats may still retain some of these mechanisms, as evidenced by the presence of histamine-inactivating enzymes in the gastrointestinal tract of these animals [51]. In some cases, this may explain the lack of symptoms following the ingestion of food containing biogenic amines, at least below a certain concentration. On the other hand, it was also reported that, in small doses, biogenic amines are quite stimulating to appetite and gut development [52].

Collectively, these studies suggest that it is unlikely that pet foods examined in the present research contain sufficient histamine to be directly toxic to cats or dogs, since the highest concentration detected was 48.3 mg/kg (sample 7 after exposure to high temperatures for 10 days), a value that is slightly lower than even the most restrictive limit set for histamine by the FDA (50 mg/kg).

Compared to pet foods, there is much more data available on the presence of histamine in foods for human consumption. Similarly, unlike in the case of pets, there is a large amount of data on histamine intoxication in humans. In 2020, official controls conducted in different EU member states showed that, in fishery products from fish species associated with a high amount of histidine, 70.42% and 73.24% of the samples collected at the distribution and manufacturing level, respectively, had a histamine concentration ≤100 mg/kg. In the same year, at the EU level, 43 outbreaks of “histamine and scombrotoxin” were reported causing 183 cases, 17 hospitalizations and 1 death [53]. The temporal trend from 2010–2020 shows a near-steady upward trend in the number of outbreaks in 2017, when there were 117 outbreaks, a decrease in 2018 (80 outbreaks) and a slight increase in 2019 (96 outbreaks) [53,54,55,56]. The significant decrease registered in 2020 may, in part, be due to the lockdown measures to fight the COVID-19 pandemic that resulted in the suspension of catering activities in restaurants and school and workplace canteens [56].

The scientific literature reports only few studies on the research of biogenic amines in pet foods. A survey conducted on a range of North American commercial pet foods and pet-food ingredients reported the presence of histamine in all samples at concentrations ranging from 3.8 to 65.5 µg/g. The study also investigated the change in histamine content of open cans of pet food stored in the refrigerator or at room temperature. In the first case, most samples showed a slightly decreased histamine content after 3 days (5 out of 9 samples) and after 7 days (7 out 9 samples). Similarly, after storage at room temperature, the samples showed slightly decreased histamine content after 3 days (7 out of 9 samples) and after 7 days (5 out 9 samples), and the sampling variation was determined to be less than 15% [49]. Guyara et al. investigated the histamine content in different fish-based cat foods, and the average concentrations detected were 11 and 23 µg/g in moist and semi-moist cat foods, respectively, while the concentrations levels ranged from 3.8 to 88.8 µg/g [57]. In a survey conducted in Austria, the occurrence of some biogenic amines was evaluated in 55 samples of canned pet (dog and cat) food, some of which contained fish. Several batches of two frozen raw materials (fish) were also sampled. The histamine concentrations detected in pet foods ranged from below the detection limit (2 mg/kg) to 94.4 mg/kg, while the median, mean and standard deviation were 12.8, 14.2 and 15 mg/kg, respectively. The analytical results also showed that amine concentrations in non-fish components were lower than in fish components for pet food, resulting in a “dilution” effect [58]. In a more recent study, Paulsen et al. determined the contents of biogenic amines and polyamines in a variety of canned foods for dogs (*n* = 72) and cats (*n* = 114) on the Austrian market. Focusing on histamine, the median and maximum contents were 25.7 and 52.2 mg/kg in dog food, and 8.4 and 61.6 mg/kg in cat food. The study showed significantly higher amounts of histamine in dog food, and these data were not expected when fish would be considered as a primary risk ingredient, since fish was not included in dog-food formulations. In canned cat food, the “fish” ingredient was identified as a statistically significant risk factor due to higher contents of cadaverine in food samples containing fish [59]. Another recent study evaluated the presence of biogenic amines in the raw materials used for dry-pet-food production. The results show that fresh meats have a significantly lower quantity of histamine compared to meat meals, and this is also the case for all the other biogenic amines assessed (cadaverine, tyramine, tryptamine, 2-phenethylamine). According to the authors, the explanation might be the microbial degradation processes that meat meal undergoes as a result of aggressive industrial processes and incorrect handling and storage conditions. The histamine concentrations detected were all below 50 mg/kg, and the mean concentrations detected in salmon meat meal and fresh meat were higher than in chicken and salmon meat meal and fresh meat [41].

## 4. Conclusions

In conclusion, the results obtained from the present study would seem to indicate a low risk of histamine intoxication in the cat foods tested in this research. However, it should be highlighted that the lack of knowledge of the factors that, together with histamine, contribute to produce mackerel syndrome and the several factors that may influence the production of histamine. In the evaluation of the risk of histamine intoxication in cats, it is also necessary to consider the greater or lesser individual sensitivity related to the enzymatic endowment of the subject or to particular health conditions, as in the case of elderly or pregnant animals. Unlike what occurs in humans, where the intake of food contaminated by histamine is generally an occasional event, pets fed with industrial food may be subject to a repeated intake. The lower percentage of positive samples and the lower level of contamination of high-end foods would seem to suggest that these products are safer than the other categories examined. It would be interesting to extend the survey to a larger number of samples to verify this finding. Furthermore, the increase in the number of cats living in apartments and the increase in pathologies, such as allergies and food intolerances in these animals, are elements to be considered for a better understanding of the impact that the presence of histamine in food can have on the health of pets. Further investigations would also be needed regarding the sensitivity of cats to histamine and the doses at which the amine can begin to induce symptomatology.

## Figures and Tables

**Table 1:** Mass spectrometric parameters for the determination of histamine using an electrospray interface (ESI) in positive ionization mode.

Analyte	MW (g/mol)	Retention Time (min)	Precursor Ion (*m*/*z*)	Product Ions (*m*/*z*)	CE (eV)
Histamine	111.15	1.8	112.0	68.1	20
				95.2 *	10

* Quantification ion.

**Table 2:** Recovery data of the method for analysis of histamine in pet-food samples spiked at 3 concentration levels.

	Histamine Spiking Level (mg/kg)			M ^2^
	20	100	200	
Recovery (%) ^1^	82.92	80.75	78.75	80.81

^1^ Average of 18 replicates for each concentration; ^2^ average recoveries of the 3 spiking levels.

**Table 3:** Results of repeatability and reproducibility tests (expressed as RSD%) calculated for analysis of histamine in pet food.

Histamine Spiking Level (mg/kg)	Repeatability			Reproducibility		
	Mean (mg/kg)	SD ^1^ (mg/kg)	RSD ^2^ (%)	Mean (mg/kg)	SD ^1^ (mg/kg)	RSD ^2^ (%)
20	16.02	1.53	9.55	16.58	1.73	10.43
100	71.02	6.34	8.93	80.75	10.55	13.07
200	148.68	13.07	8.79	157.51	13.92	8.84

^1^ Standard deviation; ^2^ relative SD.

**Table 4:** Pet-food samples and histamine concentrations.

Ref.	Product Name ^1^	Histamine (mg/kg)	Ref.	Product Name ^1^	Histamine (mg/kg)
[1]	Paté with natural tuna	<LOQ	[30]	Tuna in jelly	2.3
[2]	Chunks with tuna	<LOQ	[31]	Mackerel with seaweed	<LOQ
[3]	Chunks with tuna	<LOQ	[32]	Tuna for kittens	<LOQ
[4]	Minced tuna	<LOQ	[33]	Sardines with shrimps	<LOQ
[5]	Tuna	<LOQ	[34]	Sardines with white fish	<LOQ
[6]	With tuna	28.7	[35]	Tuna with rice	<LOQ
[7]	Tuna	29.6	[36]	With tuna	<LOQ
[8]	Tuna Japan style	<LOQ	[37]	With tuna	<LOQ
[9]	Tuna	<LOQ	[38]	With tuna	<LOQ
[10]	Pacific tuna in jelly	<LOQ	[39]	Chunks with grilled tuna	<LOQ
[11]	Tuna fillet in water and rice	<LOQ	[40]	Tuna with mackerel	<LOQ
[12]	Natural tuna	<LOQ	[41]	Tuna with mackerel	<LOQ
[13]	Chunks with trout	30.1	[42]	Tuna	<LOQ
[14]	Mackerel	<LOQ	[43]	Savory cake with tuna	<LOQ
[15]	Mousse with oceanic fish	<LOQ	[44]	With tuna fillets and anchovies	4.3
[16]	Tuna with rice	<LOQ	[45]	Tuna	<LOQ
[17]	Mackerel	<LOQ	[46]	Tuna and vitamin premix	<LOQ
[18]	Tonggol tuna fillet	<LOQ	[47]	Tuna with aloe	<LOQ
[19]	Skipjack tuna fillet	<LOQ	[48]	Pacific tuna	<LOQ
[20]	Tuna fillet and seaweed	<LOQ	[49]	Tuna	13.3
[21]	With tuna	1.5	[50]	Tuna	<LOQ
[22]	Pink tuna with mackerel	<LOQ	[51]	Chunks with trout	<LOQ
[23]	Pacific tuna with ocean fish	3.9	[52]	Chunk with oceanic fish	<LOQ
[24]	Pink tuna	<LOQ	[53]	With tuna	20.2
[25]	Tuna with rice and green beans	<LOQ	[54]	With sardines in jelly	10.8
[26]	Tuna with rice	<LOQ	[55]	With tuna	11.9
[27]	Tuna	<LOQ	[56]	With mackerel in jelly with tomato	<LOQ
[28]	Tuna with vegetables	<LOQ	[57]	With tuna in sauce	2.3
[29]	Mackerel in water	<LOQ	[58]	With tuna fillets and anchovies	<LOQ

^1^ As stated on the label on the basis of ingredients. How ingredients may be included in the product name depends on the percentage of that ingredient in the product and the use of certain descriptors.

**Table 5:** Occurrence of histamine in pet-food samples grouped on the basis of the ingredients of animal origin.

Samples	N° Positive/ N° Total Samples	% Positive	Mean (mg/kg)	Median (mg/kg)	Standard Deviation (mg/kg)	Range (mg/kg)
Pet foods containing fish (all samples)	12/58	20.7	13.2	11.4	11.2	1.5–30.1
Pet foods containing only fish ^1^	5/38	13.2	10.2	4.3	11.8	1.5–29.6
Pet foods containing tuna	10/46	21.7	11.8	8.1	11.0	1.5–30.1
Pet foods containing only tuna ^2^	3/23	13.0	15.1	13.3	13.7	2.3–29.6
Pet foods containing meat ^3^	7/20	35.0	15.4	11.9	11.2	2.3–30.1
Pet foods containing meat and tuna	5/14	35.7	13.4	11.9	11.1	2.3–28.7
Pet foods not containing meat or tuna	0/6	0.0	-	-	-	-

^1^ Products without meat or meat by-products among the ingredients of animal origin; ^2^ no other type of fish or meat among the ingredients of animal origin; ^3^ products containing mixed meat and fish or their by-products.

**Table 6:** Occurrence of histamine in pet-food samples by price range.

Samples	N° Positive/ N° Total Samples	% Positive	Mean (mg/kg)	Median (mg/kg)	Standard Deviation (mg/kg)	Range (mg/kg)
High-end products	5/32	15.6	5.1	3.9	4.7	1.5–13.3
Mid-range products	5/14	35.7	20.5	20.2	9.3	10.8–30.1
Low-end products	2/12	16.7	15.5	15.5	18.7	2.3–28.7

**Table 7:** Pet-food samples and histamine concentrations after storage in different environmental conditions.

Ref.	Product Name ^1^	Storage Conditions ^2^ and Histamine Concentrations (mg/kg)							
		T_0_-A	T_0_-B	T_1_-A	T_1_-B	T_2_-A	T_2_-B	T_3_-A	T_3_-B
[6]	With tuna	28.7	12.9	11.9	12.9	19.3	40.6	9.1	8.6
[7]	Tuna	29.6	17.2	14.6	16.9	18.3	48.3	10.2	8.4
[13]	Chunks with trout	30.1	8.2	8.8	11.6	14.3	21.7	6.8	6.9
[21]	With tuna	1.5	<LOQ	<LOQ	<LOQ	3.3	<LOQ	<LOQ	<LOQ
[23]	Pacific tuna with ocean fish	3.9	<LOQ	<LOQ	<LOQ	2.3	1.4	<LOQ	<LOQ
[30]	Tuna in jelly	2.3	<LOQ	<LOQ	<LOQ	<LOQ	2.8	<LOQ	<LOQ
[44]	With tuna fillets and anchovies	4.3	<LOQ	<LOQ	<LOQ	11.4	4.6	<LOQ	/
[49]	Tuna	13.3	5.0	6.2	6.2	20.2	31.2	5.2	5.0
[53]	With tuna	20.2	7.9	7.1	7.4	19.0	22.3	9.0	7.0
[54]	With sardines in jelly	10.8	2.7	<LOQ	3.0	13.7	18.8	2.6	1.8
[55]	With tuna	11.9	3.2	<LOQ	2.9	9.5	11.2	3.2	1.6
[57]	With tuna in sauce	2.3	<LOQ	<LOQ	<LOQ	<LOQ	<LOQ	<LOQ	<LOQ

^1^ As stated on the label on the basis of ingredients. ^2^ T_0_-A: room temperature until opening (initial control condition); T_0_-B: freezing temperature (control condition); T_1_-A: room temperature for 24 h; T_1_-B: room temperature for 48 h; T_2_-A: high temperature for 24 h; T_2_-B: high temperature for 48 h; T_3_-A: refrigerator temperature for 5 days; T_3_-B: refrigerator temperature for 10 days.

**Table 8:** Statistical analysis of the data for histamine concentrations in pet-food samples after storage in different environmental conditions.

	Groups of Samples Stored in Different Environmental Conditions and Histamine Concentrations (mg/kg)							
	T_0_-A	T_0_-B	T_1_-A	T_1_-B	T_2_-A	T_2_-B	T_3_-A	T_3_-B
Mean	13.2	5.0	4.3	5.3	11.0	17.0	4.1	3.8
Median	11.4	3.0	0.5	3.0	12.6	15.0	2.9	1.8
Standard deviation	11.2	5.6	5.2	5.7	7.7	16.4	3.8	3.4
Range	1.5–30.1	<LOQ-17.2	<LOQ-14.6	<LOQ-16.9	<LOQ-20.2	<LOQ-48.3	<LOQ-10.2	<LOQ-8.6

Experimental groups: T_0_-A = samples stored at room temperature until opening (initial control condition); T_0_-B = samples stored at freezing temperature (control group); T_1_-A = samples stored at room temperature for 24 h; T_1_-B = samples stored at room temperature for 48 h; T_2_-A = samples stored at high temperature for 24 h; T_2_-B = samples stored at high temperature for 48 h; T_3_-A = samples stored at refrigerator temperature for 5 days; T_3_-B = samples stored at refrigerator temperature for 10 days. For statistical purposes, values < LOQ were conventionally considered equal to ½ LOQ (0.5 mg/kg).

**Table 9:** Statistical analysis using t-test applied to the mean histamine concentration detected in the various group of samples stored in different environmental conditions.

Groups	T_1_-A	T_1_-B	T_2_-A	T_2_-B	T_3_-A	T_3_-B
T_0_-B	NS	NS	*	*	NS	NS
T_1_-A	-	NS	*	*	NS	NS
T_1_-B	NS	-	*	*	NS	NS
T_2_-A	NS	NS	-	NS	*	*
T_2_-B	NS	NS	NS	-	*	*
T_3_-A	NS	NS	NS	NS	-	NS

*: *p* < 0.05; NS: not significant; Experimental groups: T_0_-A = samples stored at room temperature until opening (initial control condition); T_0_-B = samples stored at freezing temperature (control group); T_1_-A = samples stored at room temperature for 24 h; T_1_-B = samples stored at room temperature for 48 h; T_2_-A = samples stored at high temperature for 24 h; T_2_-B = samples stored at high temperature for 48 h; T_3_-A = samples stored at refrigerator temperature for 5 days; T_3_-B = samples stored at refrigerator temperature for 10 days.

## Data Availability

The data presented in this study are available in the article.
